# Parallel evolution of trehalose production machinery in anhydrobiotic animals via recurrent gene loss and horizontal transfer

**DOI:** 10.1098/rsob.200413

**Published:** 2021-07-14

**Authors:** Yuichiro Hara, Reira Shibahara, Koyuki Kondo, Wataru Abe, Takekazu Kunieda

**Affiliations:** ^1^ Research Center for Genome and Medical Sciences, Tokyo Metropolitan Institute of Medical Science, Tokyo, Japan; ^2^ Department of Biological Sciences, Graduate School of Science, The University of Tokyo, Tokyo, Japan; ^3^ Department of Biology, Dokkyo Medical University, Tochigi, Japan

**Keywords:** anhydrobiosis, TPS–TPP, parallel evolution, trehalose, tardigrade, horizontal gene transfer

## Abstract

Trehalose is a versatile non-reducing sugar. In some animal groups possessing its intrinsic production machinery, it is used as a potent protectant against environmental stresses, as well as blood sugar. However, the trehalose biosynthesis genes remain unidentified in the large majority of metazoan phyla, including vertebrates. To uncover the evolutionary history of trehalose production machinery in metazoans, we scrutinized the available genome resources and identified bifunctional trehalose-6-phosphate synthase-trehalose-6-phosphate phosphatase (TPS–TPP) genes in various taxa. The scan included our newly sequenced genome assembly of a desiccation-tolerant tardigrade *Paramacrobiotus* sp. TYO, revealing that this species retains TPS–TPP genes activated upon desiccation. Phylogenetic analyses identified a monophyletic group of the many of the metazoan TPS–TPP genes, namely ‘pan-metazoan’ genes, that were acquired in the early ancestors of metazoans. Furthermore, coordination of our results with the previous horizontal gene transfer studies illuminated that the two tardigrade lineages, nematodes and bdelloid rotifers, all of which include desiccation-tolerant species, independently acquired the TPS–TPP homologues via horizontal transfer accompanied with loss of the ‘pan-metazoan’ genes. Our results indicate that the parallel evolution of trehalose synthesis via recurrent loss and horizontal transfer of the biosynthesis genes resulted in the acquisition and/or augmentation of anhydrobiotic lives in animals.

## Graphical abstract

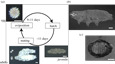

## 1. Introduction

Trehalose is a non-reducing disaccharide that potently protects various biomolecules from abiotic stresses, such as desiccation [1]. While a variety of organisms have the trehalose biosynthesis pathway, many metazoans (including vertebrates) lack the ability to produce trehalose [2]. In insects, trehalose is used as a common blood sugar [3], and some desiccation-tolerant invertebrates accumulate trehalose upon desiccation to withstand an almost complete dehydration by entering a metabolically inactive state called anhydrobiosis [4–11].

There are several pathways known to synthesize trehalose, but most of them are found only in bacteria, archaea and fungi. The trehalose-producing metazoans employ the pathway composed of trehalose-6-phosphate synthase (TPS; EC 2.4.1.15) and trehalose-6-phosphate phosphatase (TPP; EC 3.1.3.12) (electronic supplementary material, figure S1) [2,12]. To date, in metazoans, the presence of TPS, TPP or bifunctional TPS–TPP genes has been documented only for limited phyla, such as arthropods [13,14], nematodes [15], rotifers [16,17] and tardigrades [18], all of which contain anhydrobiotic species. Several studies have suggested that in metazoans, these trehalose synthesis genes were acquired via horizontal gene transfer, which was mainly proposed based on their sequence similarity to non-metazoan homologues [16,18,19]. However, the scattered information of gene retention makes it difficult to discriminate the TPS–TPP genes of non-metazoan origin from those that are derived from the common metazoan ancestors. Furthermore, sequencing experiments and analyses without ensuring the elimination of contaminants can result in the false discovery of contaminant-derived homologues as horizontally transferred genes and confound understanding of the origins and distributions of the target genes [20]. The retention and evolutionary origin of TPS–TPP genes also remain elusive in the phylum Tardigrada, which is renowned for extreme resilience against various environmental stresses, including desiccation. Among the tardigrades examined so far, the genuine TPS–TPP gene is found at least in one highly tolerant species belonging to superfamily Hypsibioidea, *Ramazzottius varieornatus,* whose genome sequence is nearly completely free from contamination [18]. Transcriptome analysis of *R*. *varieornatus* indicated no significant changes in TPS–TPP gene expression during entry to anhydrobiosis [18,21]. By contrast, *Hypsibius dujardini* (recently described as a new species, *H*. *exemplaris* [22]), another Hypsibioidea tardigrade whose genomic resource is publicly available, does not include TPS–TPP homologues in the genome assembly [21]. The tardigrade species belonging to another superfamily, Macrobiotoidea, are reported to accumulate trehalose during anhydrobiosis, and up to now no tardigrades in other taxa have been reported to show significant production of trehalose [6–8]. Genomic information of the species of Macrobiotoidea, however, is not yet publicly available. The evidence of trehalose production is patchy even in the phylum Tardigrada, which led us to investigate the retention and use of TPS–TPP genes in the phylum Tardigrada.

**Figure 1.**
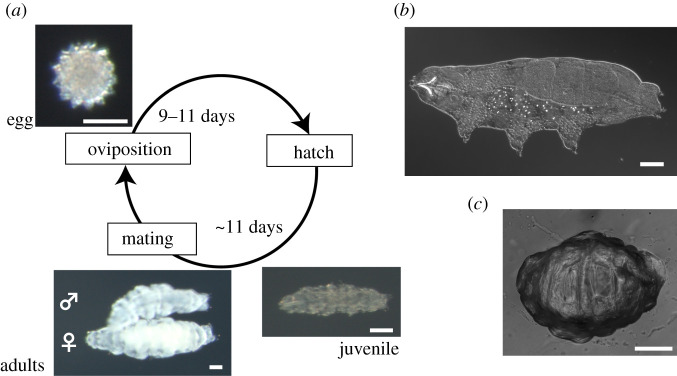
*Paramacrobiotus* sp. TYO strain. (*a*) The life cycle of the *Paramacrobiotus* sp. TYO strain. The period of each component of the cycle is in accordance with electronic supplementary material, figure S3. (*b,c*) Adult specimens in a hydrated state (*b*) and dehydrated tun state (*c*). Bars indicate 50 µm.

To clarify the evolution of trehalose production in association with anhydrobiosis in tardigrades, we performed whole-genome sequencing of the terrestrial tardigrade *Paramacrobiotus* sp. (figure 1) as well as temporal transcriptomic analysis during entry into anhydrobiosis. Whole-genome-based searches for TPS–TPP genes allowed us to distinguish them from contaminants by multifaceted evaluations. Furthermore, we intensively searched for TPS–TPP genes in metazoans to examine the extent of the sparse distribution of TPS–TPP genes in the taxonomic space. The subsequent molecular phylogenetic analyses of TPS–TPP and TPS genes led to clarification of the origin and evolution of these cryptic genes in metazoans.

## 2. Results

### 2.1. Establishment of a tardigrade strain that produces trehalose

We isolated tardigrades from bamboo leaf litter in Tokyo and identified the individuals belong to the genus *Paramacrobiotus* by examining the morphological characteristics of the adults and eggs with a phase contrast and differential interference contrast microscope (electronic supplementary material, figure S2a,b and text). This tardigrade belongs to the superfamily Macrobiotoidea, which includes a large number of species that accumulate trehalose upon desiccation [6–8]. To facilitate molecular and physiological analyses on trehalose synthesis in tardigrades, we propagated the tardigrades from a single male–female pair and established a strain dubbed *Paramacrobiotus* sp. TYO that harboured a generation time of at least three weeks (figure 1*a*; electronic supplementary material, figure S3). This taxonomic assignment was further confirmed by a molecular phylogenetic analysis (see the next section).

**Figure 2.**
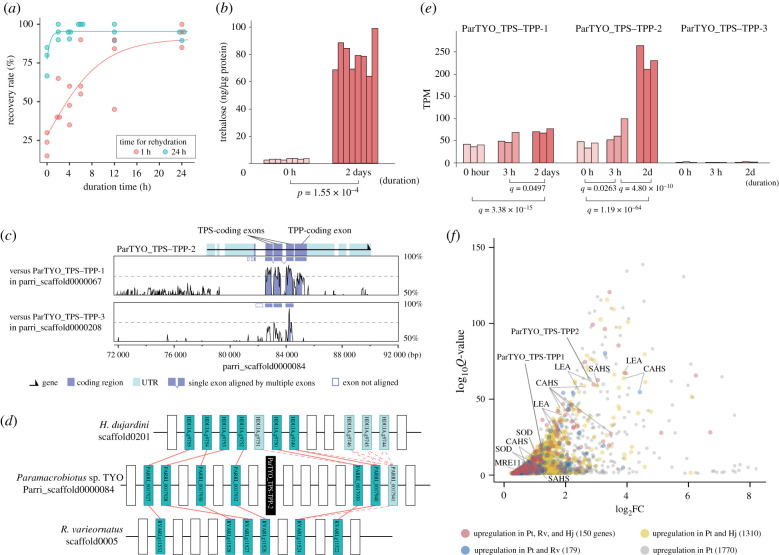
Characterization of the *Paramacrobiotus* sp. TYO trehalose biosynthesis system in the initiation of anhydrobiosis. (*a*) Longer slow-dehydration treatment enhanced the rapid recovery from desiccation. The proportion of active individuals after 1 h rehydration positively correlated with the duration of the slow-dehydration treatment (Spearman's rank correlation coefficient *r* = 0.790, *p*-value = 1.85 × 10^−5^). After 24 h of rehydration, the recovery rates were high (more than 60%) regardless of the duration time of the slow-dehydration treatment, suggesting that the short slow-dehydration groups can recover but need a longer time to recover upon rehydration. Lines indicate the fitting curves based on the sigmoid Gompertz growth model for the two rehydration conditions. (*b*) Accumulation of trehalose in the transition to anhydrobiosis. Trehalose content was quantified for the samples under the hydrated state and the dehydrated state with two days of slow-dehydration. The difference in the trehalose amounts was evaluated using Wilcoxon's rank sum test. (*c*) Genomic structure of the TPS–TPP genes and alignment of the genomic regions including these genes. The upper and lower panels indicate similarity plots between ParTYO_TPS–TPP-2 and ParTYO_TPS–TPP-1, and between ParTYO_TPS–TPP-2 and ParTYO_TPS–TPP-3. The untranslated regions of ParTYO_TPS–TPP-2 were annotated using the RNA-seq mapping-oriented transcript model generated for gene prediction. Boxes within the panels indicate the aligned and unaligned exons of ParTYO_TPS–TPP-1 and ParTYO_TPS–TPP-3 to ParTYO_TPS–TPP-2. The plots were produced by mVISTA with 50-bp windows following visualization by the VISTA browser [23]. (*d*) Synteny block retained by the three tardigrades. ParTYO_TPS–TPP-2 (black rectangle) is included in the synteny block. Solid red lines represent reciprocal BlastP best-hits between genes of two species, and dashed red lines indicate other similarity hits. Open rectangles indicate the genes harbouring no similarity hits within the synteny block. (*e*) Temporal change in the expression levels of the three *Paramacrobiotus* TPS–TPP genes over the course of the slow-dehydration treatment. *Q*-values lower than the significance level (*α* = 0.05) for the differential expression analysis between two groups are shown. (*f*) A volcano plot of the upregulation genes in the 2 d samples compared with the 0h samples. The points emphasized by gene names are trehalose synthesis genes or those specifically expanded in Tardigrada. The coloured points represent the genes retaining the orthologues of other tardigrades that are upregulated in the slowly dehydrated samples. Pt, Rv and Hj denote *Paramacrobiotus* sp. TYO, *R*. *varieornatus* and *H. dujardini*, respectively.

The TYO strain exhibited desiccation tolerance. Upon desiccation, the tardigrades gradually lose water and tolerate desiccation by entering into a reversible dehydrated state, known as anhydrobiosis, with a contracted form called the ‘tun’ (figure 1*b,c*). High-humidity conditions can slow down the rate of dehydration, which fulfils the better transition to anhydrobiosis and enables rapid recovery upon rehydration (figure 2*a*), as in other desiccation-tolerant animals [5,24–27]. This ‘slow-dehydration’ treatment invokes a gradual loss of water, offering the response time for the tardigrades to prepare protectant molecules against upcoming severe desiccation. During the 2 days of slow-dehydration treatment, the amount of trehalose in the tardigrades increased 24 times on average (figure 2*b*), indicating endogenous trehalose biosynthesis. Our results suggest that the TYO strain uses trehalose as potential protectant molecules that provide effective defence against dehydration.

### 2.2. Identification of the *Paramacrobiotus* sp. TYO trehalose synthesis genes through whole-genome sequencing

To identify the genetic backbone that allows the *Paramacrobiotus* sp. TYO strain to synthesize trehalose, we performed whole-genome sequencing. Coordination of Oxford Nanopore long-read sequencing (electronic supplementary material, figure S4) with Illumina short-read sequencing produced a highly continuous haploid genome assembly. This assembly was 170 Mb, comprising 684 scaffolds, and the scaffold N50 length was 1.03 Mb. Furthermore, assessments based on gene coverage displayed high completeness with low redundancy for the *Paramacrobiotus* sp. TYO genome assembly comparable to those for the *R*. *varieornatus* and *H. dujardini* genome assemblies (table 1). Based on the assembled genome sequences, we generated a gene model by using RNA-seq reads as hints, predicting 24 903 protein-coding genes (table 1). From the genome assembly, we also identified the sequences of 18S rRNA and mitochondrial cytochrome c oxidase I (COI) gene, both of which are frequently used for phylogenetic studies. A molecular phylogenetic analysis of 18S rRNA nucleotide sequences illuminated the affinity of this tardigrade to the genus *Paramacrobiotus* (electronic supplementary material, figure S2c). On the other hand, the COI sequence, a common DNA barcoding marker for species identification, of this tardigrade showed only moderate similarity (78–81%) to those in the other *Paramacrobiotus* species. These observations rationalized our taxonomic classification of the tardigrade as *Paramacrobiotus* sp. The exact species of the tardigrade will be described elsewhere.

**Table 1.** Statistics of the tardigrade genome assemblies and gene models.

	*Paramacrobiotus* sp. TYO	*R. varieornatus* (Rv101) [18]	*H. dujardini* (nHd.3.0.0) [21]
*genome assembly*			
assembly size	170 483 395	55 828 384	104 154 999
number of scaffolds	684	199	1421
longest scaffold length	4 484 941	9 333 084	2 115 976
shortest scaffold length	553	1000	1000
N50 scaffold length	1 033 707	4 740 345	342 180
GC content (%)	43.46	47.51	45.46
N content (%)	0.0000639	0.75	2.06
repeat element content (%)	29.75	—	—
CEGMA + CEG, complete (%)	96.77	97.58	97.98
CEGMA + CEG, partial (%)	97.98	98.39	98.79
CEGMA + CEG, no. orthologues	1.24	1.16	1.19
BUSCO + metazoan set, complete (%)	83.84	85.17	84.97
BUSCO + metazoan set, partial (%)	85.28	87.22	87.22
BUSCO + metazoan set, no. orthologues	1.05	1.02	1.02
*gene model*			
number of coding genes	24 903	13 992	19 902
number of coding transcripts	26 310	14 532	20 816
BUSCO + metazoan set, complete (%)	89.37	89.37	90.7
BUSCO + metazoan set, partial (%)	92.13	92.23	93.15
BUSCO + metazoan set, #orthologues	1.10	1.04	1.05

Within the predicted gene set, we identified three TPS–TPP genes, two of which, ParTYO_TPS–TPP-1 (gene id, PARRI_0016244) and ParTYO_TPS–TPP-2 (PARRI_0017934), encode fusion proteins of TPS and TPP, and comprised five coding exons. The third, ParTYO_TPS–TPP-3 (PARRI_0023190), contains only the TPS domain coded in four exons and no TPP domain (figure 2*c*). ParTYO_TPS–TPP-2 was located within a synteny block that was conserved in the *R*. *varieornatus* and *H*. *dujardini* genome assemblies (figure 2*d*), indicating that this gene is included in the genuine *Paramacrobiotus* sp. TYO genome rather than derived from contaminating organisms. The inclusion of all the TPS–TPP genes within the *Paramacrobiotus* sp. TYO genome was supported by the considerable depth of the mapped short reads (electronic supplementary material, figure S5a).

The inclusion of all the TPS–TPP genes within the TYO genome was further verified by identifying their putative orthologues in the transcriptome assembly of the closely related species *P. richtersi* that inhabits leaf litter in Italy [28,29] (hereafter referred to as ITA). We created this transcript assembly *de novo* by employing the RNA-seq reads retrieved from the public repository (see electronic supplementary material). For the confirmation of the orthologies of the three TPS–TPP genes between TYO and ITA, we computed the numbers of transversions for fourfold degenerate sites (4DTv), a proxy for a neutral nucleotide substitution rate, for all the one-to-one orthologues including the three TPS–TPP genes between the two strains. The 4DTv values of the three TPS–TPP genes are within the confidence interval of those of all the one-to-one orthologues (electronic supplementary material, figure S5d and text), strongly indicating the genuine orthologies of the three TPS–TPP genes.

We further performed a more sensitive search for TPS–TPP homologues by employing Delta-BLAST [30], a protein domain-aware similarity search with high sensitivity that allows to detect distant homologues, followed by an iterative PSI-BLAST [31]. This search did not detect any additional genes encoding TPS and/or TPP domains. In a similar way, we conducted a thorough search for the homologues of the trehalose synthesis isozymes, TreY (EC 5.4.99.15), TreZ (EC 3.2.1.141), TreP (EC 2.4.1.64), TreS (EC 5.4.99.16) and TreT (EC 2.4.1.245), all of which are not homologous to TPS–TPP. We did not find any homologues of them in metazoans, indicating that *Paramacrobiotus* tardigrades, as well as other metazoans, employed only the TPS–TPP genes for endogenous trehalose synthesis.

### 2.3. Characterization of *Paramacrobiotus* TPS–TPP genes and exploration of their origin

To examine whether the expression of TPS–TPP genes is associated with trehalose production for the *Paramacrobiotus* sp. TYO, we performed transcriptome analysis using three samples: no-dehydration treatment (0 h), and slow-dehydration treatment for three hours (3 h) and two days (2 d). The differential expression analysis identified 1053 genes between the 0 h and 3 h samples, 6391 genes between the 0 h and 2 d samples, and 3828 genes between the 3 h and 2 d samples (electronic supplementary material, table S3). Our results indicate that two *Paramacrobiotus* sp. TYO TPS–TPP genes, ParTYO_TPS–TPP-1 and ParTYO_TPS–TPP-2, were significantly upregulated during slow-dehydration treatment (figure 2*e,f*). This upregulation of the TPS–TPP genes demonstrated a temporal concordance with the significant accumulation of trehalose (figure 2*b*), suggesting that these genes participate in the endogenous biosynthesis of trehalose. The forced expression of the most upregulated TPS–TPP gene (ParTYO_TPS–TPP-2) in human cultured cells produced a significant amount of trehalose (electronic supplementary material, figure S6), which validated the trehalose biosynthetic activity of the gene products. The upstream genomic regions of these two genes shared moderate similarity with each other (figure 2*c*), implying the utilization of similar gene regulatory circuits for their expression. Expression of the remaining gene, ParTYO_TPS–TPP-3, on the other hand, remained quite low during all the stages.

We further examined whether the *Paramacrobiotus* TPS–TPP genes were acquired from another organism, as that of *R*. *varieornatus* was. For this purpose, we computed the horizontal gene transfer (HGT) index [32], a rough but simple metric based on a similarity score, with slight modifications: we used BlastP bit scores instead of BlastX ones and, to reduce false positives, employed our unique correction weighted by alignment lengths (see electronic supplementary material). We classified 112 genes that were horizontally transferred after separation from Hypsibioidea (electronic supplementary material, table S4), including all three *Paramacrobiotus* sp. TYO TPS–TPP genes. Because the BlastP searches demonstrated that the three paralogues were more similar to each other than to any homologues of other species, a TPS–TPP gene could have been transferred into the ancestral *Paramacrobiotus* genome followed by gene duplications.

### 2.4. Early origin of genomic components associated with dehydration in tardigrades

Besides the trehalose synthesis genes, we detected tolerance-related features shared by the two previously decoded tardigrade genomes belonging to another superfamily, Hypsibioidea [18,21]. Our orthologous clustering displayed the expansions of LEA (Late embryogenesis abundant), CAHS (Cytosolic-abundant heat soluble), SAHS (Secretory Abundant Heat Soluble), SOD (Superoxide dismutase) and MRE11 (Meiotic Recombination 11) genes that are potentially involved in protection and repair of biomolecules. Previous whole-genome analyses exhibited the lineage-specific expansions of the genes in *R*. *varieornatus* and *H. dujardini* [18,21]. Transcriptome analysis further indicated that CAHS and SAHS are absent from order Heterotardigrada, but the others are retained by both Eutardigrada and Heterotardigrada [33]. The cross-species transcriptome analysis revealed that some of these genes were significantly highly expressed in the slowly dehydrated samples treated with high humidity exposure (figure 2*f*; electronic supplementary material, tables S1 and S3, and text).

In addition, the *Paramacrobiotus* sp. TYO genome lacks the genes involved in the stress-responsive mTORC1 (mechanistic target of rapamycin C1) regulatory pathway and the peroxisomal oxidative pathway, as do the other two tardigrade genomes (electronic supplementary material, table S2 and text). Our results indicate that these tolerance-related machineries were established before the split between the superfamilies Macrobiotoidea and Hypsibioidea.

### 2.5. Sporadic distribution of the TPS–TPP genes in metazoa

The claims for inferred horizontal transfer of several metazoan TPS–TPP genes, including those of *Paramacrobiotus* sp. TYO, largely rely on the absence of their closely related homologues in animals. To verify the extent of the retention of the metazoan TPS–TPP genes, which remain unidentified in many phyla, we performed an in-depth search for these genes within the publicly available genome assemblies (electronic supplementary material, table S5; 488 vertebrates, 416 arthropods, 82 nematodes and 91 other invertebrates deposited in NCBI WGS by 17 February 2017). To exclude the homologues of contaminants, our search was restricted to genes that were split into multiple exons in the genome assembly and exhibited moderate or low similarity (less than 80%) of the nucleotide sequences to those of any non-metazoan homologues. All of the TPS–TPP genes found in the arthropods and nematodes were orthologous to the well-documented genes in these phyla. In addition to these, we found a *Branchiostoma floridae* homologue harbouring the TPS domain in the RefSeq database (NCBI RefSeq peptide ID: XP_002598453.1) with transcriptional evidence by EST (electronic supplementary material, figure S5f). We further scrutinized the metazoan genome assemblies accompanying on-demand gene prediction of the TPS–TPP genes (see Material and methods). This inspection identified the TPS–TPP genes of the star ascidian (*Botryllus schlosseri*), spiny brittle star (*Ophiothrix spiculata*) and sea anemone (*Aiptasia* sp. CC7). Among them, the *O. spiculata* and *Aiptasia* sp. homologues were located in the scaffolds harbouring considerable mapping depths of short reads (electronic supplementary material, figure S5b,c), suggesting their intrinsic nature within the animal genomes. Interestingly, these metazoan homologues exhibited the BlastP best hit with the arthropod TPS–TPP genes, indicating their orthologous relationship.

Our inspection of the metazoan TPS–TPP genes extended to the transcriptome assemblies available from the public repository (electronic supplementary material, table S5; 219 invertebrates excluding arthropods and nematodes). The similarity search uncovered the TPS–TPP homologues of three annelids, bloodworm (*Glycera dibranchiata*), clamworm (*Perinereis aibuhitensis*) and hydrothermal vent tubeworm (*Lamellibrachia satsuma*), a monogonont rotifer (*Brachionus manjavacas*), and the Waratah anemone (*Actinia tenebrosa*): all of them showed the highest BlastP bit scores with the arthropod homologues. We also retrieved partial sequences of the putative orthologue of the *B*. *manjavacas* TPS–TPP gene from the EST sequences of its relative *B*. *plicatilis*, and the orthology was confirmed with the 4DTv distribution between putative orthologues of the two species (electronic supplementary material, figure S5e). To provide further insight into the origin of metazoan TPS–TPP genes, we searched for the TPS–TPP homologues in the genome and transcriptome assemblies of 17 unicellular holozoans, the closest relatives of the metazoans (electronic supplementary material, table S6), but found none (see electronic supplementary material).

### 2.6. Frequent loss and horizontal transfer of the metazoan TPS–TPP genes

**Figure 3.**
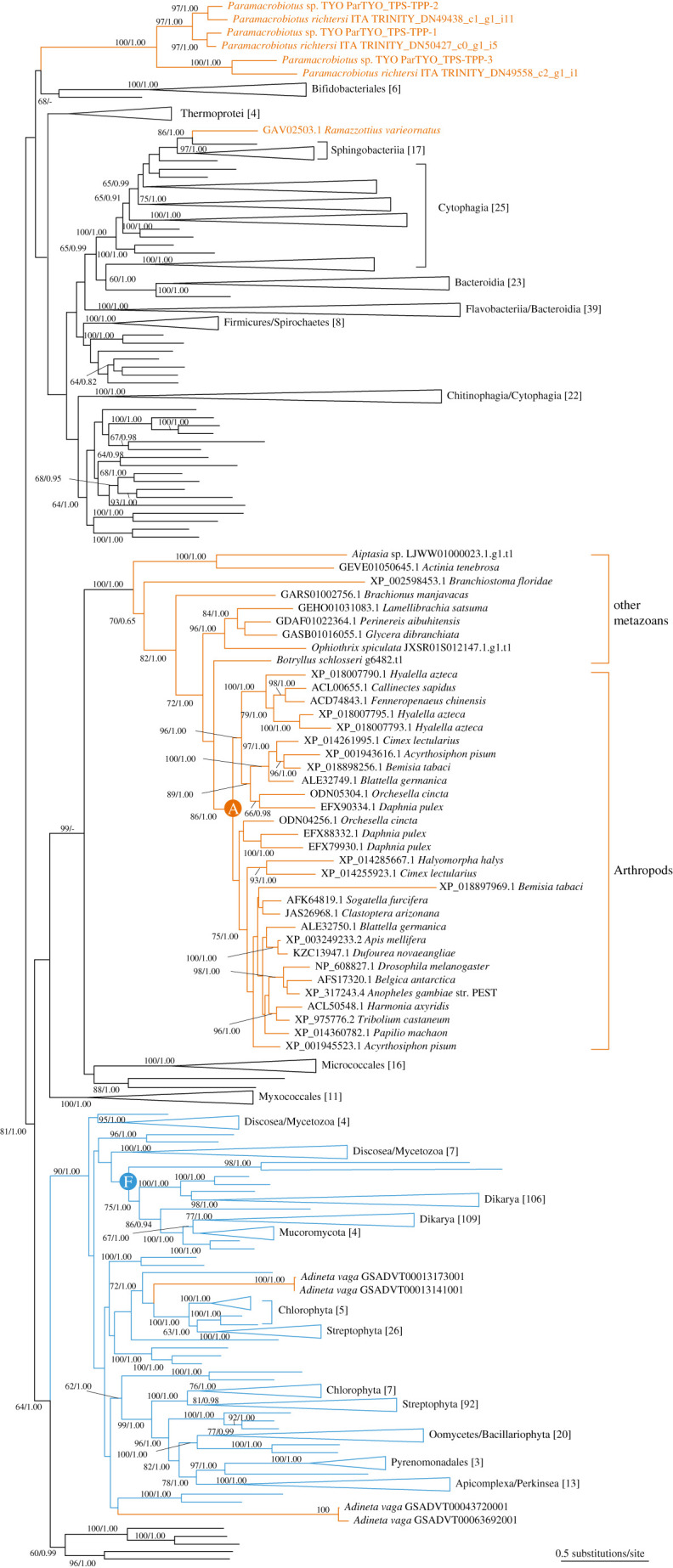
TPS–TPP gene phylogeny. The TPS–TPP gene tree inferred with the maximum-likelihood method, using 677 operational taxonomic units (OTU) containing 643 amino acid sequence alignment sites. Orange and blue branches correspond to metazoan and non-metazoan eukaryote lineages, respectively. The leaf names in orange denote the tardigrade TPS–TPP genes. If a subtree contains at least three OTUs and greater than or equal to 90% of them were occupied by a single taxon, it was collapsed into a triangle shape visualized by FigTree v. 1.4.3, and its representative taxon and number of OTUs are shown at the triangle. Bootstrap values of at least 60 are shown at the nodes, where the Bayesian posterior probabilities of 0.8 or larger are also displayed. Closed circles denoted by letters ‘A’ and ‘F’ represent the nodes of arthropods and fungi, respectively, used for the topological constraints in the tree topology tests (table 2). The intact tree topology is available in electronic supplementary material, datasets S1 and S2 for the ML and Bayesian trees, respectively.

To disentangle the evolutionary history of the metazoan TPS–TPP genes, we inferred a phylogenetic tree incorporating the homologues of both eukaryotes and prokaryotes. Both of the maximum-likelihood (ML) and Bayesian trees exhibited a monophyletic group composed of the homologues of arthropods and several metazoan phyla with a bootstrap value of 100 and the Bayesian posterior probability of 1 (figure 3; electronic supplementary material, figure S7), revealing their orthologous relationships. Hereafter, we call the genes belonging to this monophyletic group ‘pan-metazoan’ TPS–TPP genes. Besides arthropods, the pan-metazoan genes were found in only several invertebrate species, strongly indicating its loss-prone nature during evolution. The *R*. *varieornatus* TPS–TPP genes, which were distantly related to the pan-metazoan genes, were closest to those of *Solitalea canadensis*, a sphingobacterium isolated from soil. Importantly, the *Paramacrobiotus* duplicates comprised a monophyletic group distant from both the pan-metazoan and *R*. *varieornatus* TPS–TPP genes, and displayed one-to-one orthologous relationships between the TYO and ITA (figure 3) in concordance with the inferences based on the similarity searches mentioned in the previous sections (electronic supplementary material, figure S5d). Our result indicates the distinct origins of the *Paramacrobiotus* and *R*. *varieornatus* TPS–TPP genes through parallel horizontal gene transfer events within the phylum Tardigrada.

Besides the horizontally transferred genes in tardigrades, we found a few foreign genes of metazoans within the TPS–TPP gene tree. Previous literature indicated that two groups of bdelloid rotifer TPS genes, all of which lost the TPP domain, have close phylogenetic relationships to non-metazoan eukaryotes [16]. Our phylogenetic analysis also supported this phylogenetic relationship, a monophyletic group of non-metazoan eukaryotes including the bdelloid rotifer homologues (figure 3). The analysis further revealed that the bdelloid rotifer TPS genes are closely related to chlorophyte and stramenopile homologues that retain both TPS and TPP domains (figure 3), implying that the ancestor of bdelloid rotifers had acquired TPS–TPP bifunctional genes followed by loss of the TPP domain. The gene tree displayed a distant relationship between the non-metazoan eukaryotes and the pan-metazoan TPS–TPP genes, indicating the independent origin of the pan-metazoan TPS–TPP genes from the eukaryote group potentially via horizontal gene transfer. In addition, we inferred phylogenetic trees of TPS and TPP domains separately, with an increased number of OTUs owing to the inclusion of the genes harbouring the TPS or TPP solely, while the aligned sites were decreased. The ML and Bayesian trees of the individual domains again exhibited the putative horizontal transfers that were observed within the TPS–TPP trees (electronic supplementary material, figures S7–S9).

We extensively examined the validity of the suggested horizontal gene transfer events. To achieve this, we employed the tree topology test to assess if the ML tree topology harboured a significantly better explanation than the alternative topologies that did not postulate horizontal gene transfers on the basis of the likelihood values. These alternative topologies assumed monophyletic relationships between native and foreign genes concordant with the species phylogeny. The approximately unbiased (AU) test [34], which has been widely employed for this purpose [35–37], significantly rejected these native–foreign monophyletic clusterings of the pan-metazoan TPS–TPP genes and the putative horizontally transferred genes of *Paramacrobiotus*, *R*. *varieornatus* and one of the bdelloid rotifer groups (*p*-value = 3.28 × 10^−8^, 4.61 × 10^−11^ and 1.92 × 10^−5^, respectively; table 2), as well as the clustering of fungus TPS–TPP genes and the pan-metazoan genes (*p*-value = 9.34 × 10^−4^; table 2). Additionally, the monophyletic clustering of the *Paramacrobiotus* and *R*. *varieornatus* TPS–TPP genes was significantly rejected, too (*p*-value = 0.00808), clearly supporting multiple horizontal gene transfers in the individual tardigrade lineages rather than a single event in the ancestral lineage.

**Table 2.** Tree topology test for monophyletic clustering of the native and foreign genes of the individual horizontal transfer events.

domains used	tree topology constraints	−ΔlnL	*p*AU^a^	*p*KH^b^
TPS–TPP	(arthropods^c^, *Paramacrobiotus*)	250.1	3.28 × 10^−8^	<1 × 10^−5^
	(arthropods^c^, *Ramazzottius*)	559.0	4.61 × 10^−11^	<1 × 10^−5^
	(*Brachionus* rotifer, bdelloid rotifer-1^d^)	230.0	1.92 × 10^−5^	3.32 × 10^−5^
	(*Brachionus* rotifer, bdelloid rotifer-2^e^)	92.26	0.104	0.0509
	(pan-metazoans, fungi^c^)	176.8	9.34 × 10^−4^	7.06 × 10^−4^
	(*Paramacrobiotus*, *Ramazzottius*)	155.3	0.00808	0.00851
TPS	(arthropods^f^, nematodes)	71.45	0.353	0.225

^a^*p*-value of the approximately unbiased (AU) test [34].

^b^*p*-value of the Kishino-Hasegawa (KH) test [38].

^c^The nodes representing the taxonomic groups are shown in figure 3.

^d^A monophyletic group of GSADVT00013141001 and GSADVT00013173001.

^e^A monophyletic group of GSADVT00043720001 and GSADVT00063692001.

^f^The node representing the taxonomic group is shown in electronic supplementary material, figure S8.

We further scanned the multiple alignments to obtain phylogenetic signatures that distinguished the pan-metazoan genes from the homologues of the outer taxa. The precise inspection revealed an array of insertions and a deletion in the TPS domain that were specifically retained by bilaterians of the pan-metazoan genes and in parts harboured by the cnidarians, too (electronic supplementary material, figure S10). Remarkably, these signatures were not observed in any of the putative transferred genes of tardigrades and bdelloid rotifers (electronic supplementary material, figure S10), including the other bdelloid rotifer group that did not exhibit a statistical significance (*p*-value = 0.104; table 2). The result strongly suggested that these metazoan genes were individually transferred from the outer taxa.

The ML and Bayesian trees of the TPS domain indicated a horizontal gene transfer of the nematode TPS genes, to which *Caenorhabditis elegans tps-1/-2* belonged (electronic supplementary material, figure S8). Although the AU-test did not statistically reject the monophyly of the arthropods in the pan-metazoan TPS–TPP genes and the nematode TPS genes (*p*-value = 0.353; table 2), the phylogenetic signatures in the amino acid sequences explicitly discarded the inclusion of the nematodes within the pan-metazoan group (electronic supplementary material, figure S10). The nematode TPS genes also contain phosphatase domains phylogenetically proximate to that of *C*. *elegans* TPP (*gob-1*), but far from the pan-metazoan TPS–TPP genes (electronic supplementary material, figure S9 and text). The results imply that a bifunctional TPS–TPP gene was transferred into the ancestral nematode genome followed by subfunctionalization into the TPS and TPP enzymes in modern nematodes [15]. In summary, the coordination of the quantitative and qualitative assessments allowed us to evaluate the putative horizontal transfer events adequately. Our results lead to an idea that multiple origins of the TPS–TPP genes in the metazoans were shaped by the frequent loss of the pan-metazoan genes and horizontal transfer of the homologues.

### 2.7. Search for trehalase genes in *Paramacrobiotus* sp. TYO

**Figure 4.**
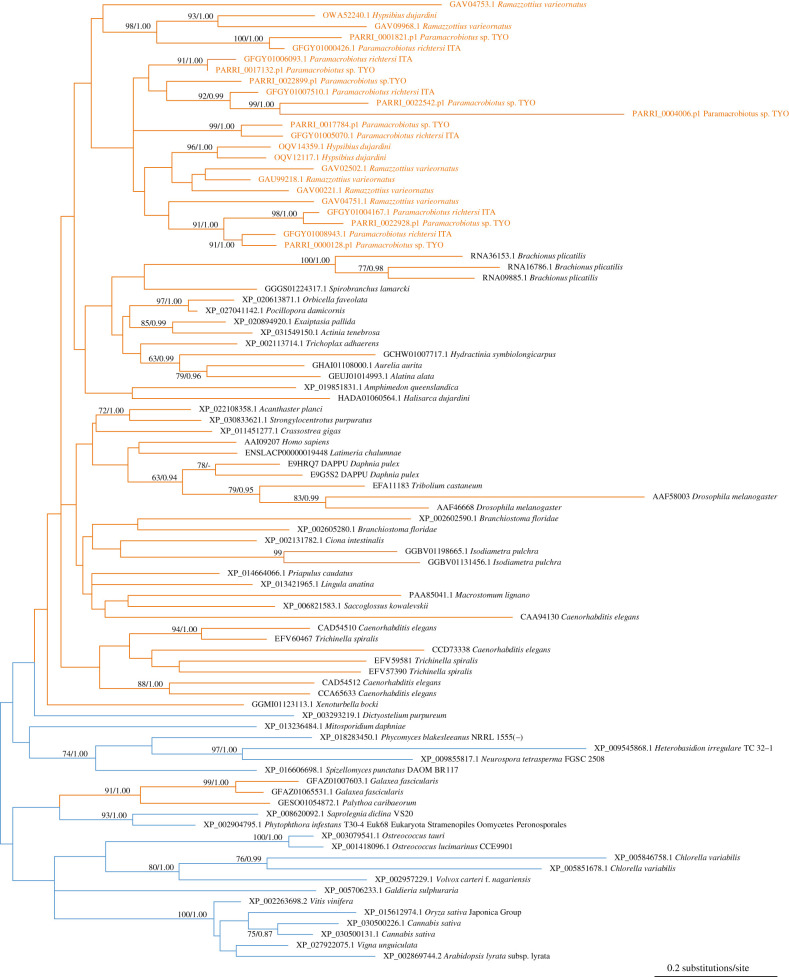
Trehalase gene phylogeny. The eukaryote trehalase gene tree inferred with the maximum-likelihood and Bayesian methods, using 88 OTUs containing 191 amino acid sequence alignment sites. The trehalase gene tree of all the three kingdoms were shown in electronic supplementary material, figure S11, and the sequences of the representative eukaryote homologues were retrieved from a eukaryote monophyletic group of the tree. See the legend in figure 3 for details of tree visualization. The intact tree topology is available in electronic supplementary material, datasets S7 and S8 for the ML and Bayesian tree, respectively.

We found eight paralogues that encode trehalase in the *Paramacrobiotus* sp. TYO genome assembly. Similarly, the *R*. *varieornatus* and *H. dujardini* genome assemblies contain six and three trehalase paralogues, respectively. Molecular phylogenetic analysis revealed that many phyla of metazoans retain trehalase genes, a large part of which shaped a monophyletic group (figure 4; electronic supplementary material, figure S11). Notably, *Paramacrobiotus* sp. TYO retains the largest number of trehalase duplicates among metazoans as far as we investigated, and the tardigrade treahalase genes were duplicated before the split between the superfamilies Macrobiotoidea and Hypsibioidea (figure 4). Since a large majority of metazoans do not harbour trehalose synthesis machinery, the trehalase genes may act as degradation of exogenous trehalose. Nevertheless, our analysis implies that these trehalase duplicates in *Paramacrobiotus* sp. TYO retain the ability for quick degradation of endogenous trehalose in recovery from anhydrobiosis.

## 3. Discussion

Horizontal gene transfer can confer phenotypic innovation, although it occurs less frequently in metazoans than in prokaryotes [39]. The identification of horizontally transferred genes within metazoan genomes requires close attention because the genes must be distinguished from the homologues of contaminating organisms that could be the host of the transferred genes. With this in mind, we identified three horizontally acquired TPS–TPP genes of the trehalose-producing tardigrade *Paramacrobiotus* sp. TYO through whole-genome sequencing rather than gene-by-gene detection. We confirmed the horizontal gene transfer by employing multifaceted validations with genomic and phylogenetic approaches, revealing the inclusion of the TPS–TPP genes in the genuine *Paramacrobiotus* sp. TYO genome assembly (figure 2*c*,*d*; electronic supplementary material, figure S5a,d) and rejecting the null hypothesis that the genes were orthologous to the ‘pan-metazoan’ genes (figure 3; table 2; electronic supplementary material, table S4). The confirmation of the horizontal gene transfer was due to the high-quality genome assembly that was accomplished by careful removal of contaminants from the animal samples. Our rearing system feeds the tardigrades on an alga *Chlorella vulgaris* and a rotifer *Lecane inermis*, potentially a source of a contaminant of the genome assembly. Similarity searches, however, found none of the sequences identical to the *Paramacrobiotus* sp. TYO TPS–TPP genes within the *C*. *vulgaris* genome assembly (NCBI Assembly ID GCA_001021125.1). The aforementioned genomic-scale assessment of contaminations was coordinated with on-demand gene prediction within the metazoan genome and transcriptome assemblies, allowing us to further identify the TPS–TPP genes of a variety of metazoan phyla (electronic supplementary material, figure S5).

**Figure 5.**
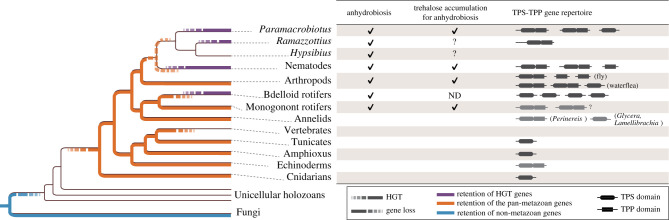
Evolutionary history of TPS–TPP genes in metazoans. Gain–loss history of the TPS–TPP genes and capability of trehalose synthesis and anhydrobiosis in modern metazoan species [5,9,10,16,25,26]. The genes shown in dark and light grey were identified within the genome and transcriptome assemblies, respectively. More gene loss events than those shown in the figure may have occurred because the homologues were identified in a limited number of metazoan species.

Our intensive search for metazoan homologues and elaborate molecular phylogenetic analyses (table 2; electronic supplementary material, figure S10) allowed us to disentangle the evolutionary history of the cryptic TPS–TPP genes in metazoans as summarized in figure 5. The eukaryotic TPS–TPP genes are likely to have been lost in early holozoans (see electronic supplementary material) following the secondary acquisition of the TPS–TPP genes, namely pan-metazoan genes, in the common ancestor of the eumetazoans at the latest. During metazoan evolution, the TPS–TPP genes were frequently lost in a variety of lineages. The tertiary acquisition of the TPS–TPP genes occurred in parallel in the lineages leading to *Paramacrobiotus*, *R*. *varieornatus*, nematodes and bdelloid rotifers, all of which include species harbouring anhydrobiotic ability. No pan-metazoan TPS–TPP genes were found in these taxa, although it is uncertain which of the two events predated the other; the loss of the pan-metazoan genes, or the horizontal acquisition of the homologues. The tertiary acquired TPS–TPPs are known to be functional at least in *Paramacrobiotus* and nematodes according to the observed accumulation of trehalose upon the initiation of anhydrobiosis. Our phylogenetic analyses indicate that all of the metazoan trehalose biosynthetic genes were transferred as bifunctional TPS–TPP and, in some of the lineages, later lost either of the catalytic domains sometimes after gene duplication.

The trehalose quantification and transcriptome analyses of the *Paramacrobiotus* sp. TYO illuminates a significant increase in trehalose in the preparation for anhydrobiosis (figure 2*b*) with upregulation of the two TPS–TPP genes (figure 2*e*). In comparison with dauer larvae of *C*. *elegans* and larvae of the sleeping chironomids for which trehalose is indispensable to survive desiccation for an anhydroprotectant role [8,25,26,40], *Paramacrobiotus* sp. TYO accumulates a much smaller amount of trehalose in both the active and dehydrated states (figure 2*b*) [5,26]. The results imply that the trehalose biosynthesis in *Paramacrobiotus* sp. TYO augments the robust formation of anhydrobiosis with low trehalose concentration. This speculation is consistent with the evolutionary process of anhydrobiosis in tardigrades: trehalose-independent anhydrobiosis could be already established in common ancestors of Macrobiotoidea and Hypsibioidea at the latest [6–8] (e.g. figure 2*f*; electronic supplementary material, tables S1 and S2). A recent study indicated that sleeping chironomids also employ trehalose as an energy source for a safe recovery from anhydrobiosis [41]. The co-occurrence of TPS–TPP acquisition and trehalase gene expansion in *Paramacrobiotus* sp. TYO implies the utilization of trehalose for the carbohydrate sources in rehydration as do sleeping chironomids.

Nematodes and sleeping chironomids, as well as *Paramacrobiotus*, commonly accumulate trehalose by employing the TPS–TPP pathway during the preparation period for anhydrobiosis triggered by exposure to highly humid conditions [5,7,8,25,26]. Most importantly, our study revealed the independent origins of the TPS–TPP pathway in these three groups through recurrent gene loss and horizontal transfer (figure 5). This indicates the parallel evolution of the trehalose accumulation for anhydrobiosis: anhydrobiosis itself could arise in parallel in multiple metazoan phyla. Multiple horizontal transfer events of TPS–TPP genes in taxa including anhydrobiotic animals seem to conflict with the currently accepted theory that horizontal gene transfer is infrequent in metazoans [21,42,43]. This prompted us to posit that horizontally acquired TPS–TPP genes have been under strong evolutionary pressure for their retention because of their contribution to anhydrobiosis. TPS–TPP genes encode a bifunctional enzyme [13] that alone confers a complete trehalose synthesis pathway, and many of the metazoans retain a trehalose-decomposing enzyme, trehalase (figure 4). Accordingly, the potentially harmful accumulation of intermediates and end products could be avoided even when the TPS–TPP pathway was horizontally acquired, which might result in a moderate force of natural selection against the gene and hence an increase of a chance of its survival. Furthermore, our rough detection of the horizontally transferred genes in the *Paramacrobiotus* sp. TYO genome indicated that newly acquired genes after the separation from the superfamily Hypsibioidea were overrepresented in the differentially expressed genes between the 0 h and 2 d samples (*p*-value = 1.68 × 10^−5^; odds ratio, 2.35; electronic supplementary material, table S4). Notably, the TPS–TPP genes, which were classified to the above ‘newly acquired’ genes, exhibited differential expression levels between the two time points (figure 2*e*). The observations imply that the incorporation into molecular machinery of anhydrobiosis makes the horizontally transferred genes less prone to loss, although intensive sequence analyses are still required to confirm the horizontal transfer events for each gene, as we did for the TPS–TPP genes. The *R*. *varieornatus* TPS–TPP gene did not exhibit a significant change in expression level during anhydrobiosis, while we validated its catalytic activity (electronic supplementary material, figure S6). The elucidation of its functional involvement in anhydrobiosis may provide further insight into the survival of the TPS–TPP genes during evolution in the anhydrobiotic tardigrades.

Our in-depth search identified putative orthologues of arthropod TPS–TPP genes within a variety of metazoan taxa that do not include anhydrobiotic species. The indispensability of the TPS–TPP genes in arthropods was demonstrated by fruit fly *Tps1* mutants [13,44]. Outside arthropods, however, the pan-metazoan TPS–TPP genes are retained by a limited number of species and absent from most of the metazoan genome and published transcriptome assemblies (e.g. four out of 91 species genome assemblies examined; electronic supplementary material, table S5). Our finding implies that in metazoans, the trehalose synthesis pathway with the TPS–TPP genes has been frequently lost. Additionally, some metazoans retain the pan-metazoan TPS–TPP orthologues that lost TPP domain (figure 5). The loss of the TPP domain could be harmful because the intermediate trehalose 6-phosphate is potentially toxic. Nematodes retain TPS and TPP coded in separate loci, TPS in *tps-1* and *tps-2* and TPP in *gob-1*, though *tps-1* and *tps-2* harbour homologous sequences to *gob-1* TPP domain (electronic supplementary material, figure S9 and text). The *gob-1* knockdown results in lethality in larvae due to the accumulation of trehalose 6-phosphate [15]. This observation indicates that the retention of only a TPS domain could have been severely disadvantageous for survival in metazoans. Accordingly, the animals that retain only a TPS domain might have acquired machinery that neutralizes the toxicity of trehalose 6-phosphate and have used the molecule for alternative purposes such as signal molecules, as observed in plants and fungi [12,45,46].

Our study uncovered inter-phyla parallel evolution of trehalose accumulation in the initiation of anhydrobiosis through horizontal transfer of the genes coding trehalose synthesis enzymes accompanying the loss of pan-metazoan homologues. Although it is certain that horizontal gene transfers in tardigrades are as infrequent as in other metazoans, our results illuminate an important role of horizontal gene transfer for the evolution of anhydrobiosis. Additionally, it is demonstrated that our genome-based gene identification allows elucidating cryptic distribution of the metazoan homologues that contains frequent loss and horizontal acquisitions with minimizing artificial effects derived from contaminations. Our approach, coordinating with the utilization of increasing sequence information of metazoan genomes, would be widely applicable to further identification of patchily distributed genes in the metazoan taxonomic space. This elaborate gene identification may give more attention to the clarification of the evolutionary history and functional significance of horizontally transferred genes and frequently lost genes, which may still remain a mystery.

## 4. Material and methods

### 4.1. Animal rearing and experiments

#### 4.1.1. Animal rearing

Terrestrial tardigrades, *Paramacrobiotus* spp*.,* were collected from bamboo leaf litter at the Tozenji temple in Tokyo, Japan (35.742983°N, 139.549757°E) in November 2006. The extracted tardigrades were propagated in a dioecious manner, and the strain termed TYO was established by mating a single male with a single female. We examined the morphological characteristics of the isolated tardigrades (adults and eggs) under a light microscope with differential interference contrast and identified them as *Paramacrobiotus* sp. TYO*;* their morphology was consistent with the descriptions of *Paramacrobiotus* in the literature [47]. For breeding, we reared the tardigrade individuals in a thin water layer on 1.2% agar plates at 22°C, and fed them a mixture of algae, *Chlorella vulgaris* (Chlorella Industry Co., Japan, or Recenttec K. K., Japan), and small monogonont rotifers, *Lecane inermis,* in a manner similar to that described for other tardigrades [48].

#### 4.1.2. Desiccation tolerance assay

Approximately 20 tardigrades in 125 µl water were dropped onto a nylon net filter (NY1002500; Merck Millipore, Germany) placed on 25-mm 3MM filter paper (GE Whatman, Pittsburgh, PA, USA). ‘Slow-dehydration’ treatment was performed according to the following procedures at 22°C. Animals were incubated in a humidity chamber (400 ml) with 95% relative humidity regulated by a saturated potassium nitrate solution for 0 h (no slow-dehydration) to 48 h. After slow-dehydration treatment, the tardigrades were desiccated at 10% relative humidity for 2 days in a humidity chamber containing silica gels. Dehydrated individuals were pre-humidified at 95% relative humidity for 1 day and then rehydrated with sterilized Milli-Q water (Merck Millipore, Germany). Their locomotion activities were examined at 1 and 24 h after rehydration: animals exhibiting spontaneous locomotion were considered to be active. This experiment was conducted with three replicates for each experimental condition varying the duration of the ‘slow-dehydration treatment (figure 2*a*).

#### 4.1.3. Quantification of trehalose contents

We measured the trehalose contents of *Paramacrobiotus* sp. TYO individuals under a hydrated condition and a dehydrated condition after 2 days of slow-dehydration treatment. Adult tardigrades were starved for a day followed by extensive cleaning and transferred into 16 low binding 1.5 ml tubes, each containing approximately 100 tardigrades in 2 µl Milli-Q water. Eight tubes were immediately frozen by liquid nitrogen and stored at −30°C as hydrated samples. The other eight were exposed to ambient air (approx. 45% relative humidity) leaving the lids open to facilitate evaporation of water. The disappearance of the water surrounding tardigrades was confirmed by visual inspection with a stereomicroscope, and then the tubes were immediately placed into a humidity chamber with 95% relative humidity and maintained for 2 days, as described in the previous section.

To prevent artificial decomposition of trehalose and proteins, we homogenized tardigrade samples quickly in ice-cold 15 µl reaction buffer (Trehalose Assay Kit, Megazyme, USA) containing protease inhibitors (cOmplete EDTA-free Protease Inhibitor Cocktail; Roche, Switzerland) on ice using a disposable plastic pestle (KONTES; Sigma, USA). The pestle was rinsed with 85 µl ice-cold reaction buffer, which was mixed with the previous homogenate (approx. 100 µl in final). A portion (approx. 80 µl) of the homogenate was heated at 80°C for 15 min to inactivate endogenous enzymes including trehalases, and subjected to trehalose quantification using fluorometric detection [49,50]. Briefly, the samples were treated with glucose oxidase (0.5U; Sigma, USA) and catalase (7.5U; Sigma, USA) at 30°C for 60 min to decompose endogenous glucose, and the reaction was stopped by heat inactivation at 80°C for 15 min. Residual glucose content was determined using Amplex Red Glucose/Glucose Oxidase Assay Kit (ThermoFisher, USA). After quantification of glucose amount, bacterial trehalase (treF; Megazyme, USA) was added to convert endogenous trehalose to twice-molar glucose, which was then quantified. Trehalose amount was determined as an increase of glucose content after the addition of trehalase. The protein content of each unheated sample was determined using Pierce Coomassie (Bradford) Protein Assay Kit (ThermoFisher, USA) for tardigrade samples or BCA protein Assay Kit (ThermoFisher, USA) for human cell extracts.

#### 4.1.4. Expression of tardigrade TPS–TPP in human cultured cells

The expression constructs were built as follows. The coding sequences of ParTYO_TPS–TPP-2 and *R. varieornatus* TPS–TPP (RvTPS–TPP, RvY_13060) were amplified from adult cDNAs of each species with the following primers; ParTYO_TPS–TPP-2 forward: 5′-TTAAACTTAAGCTTGGTACCATGCCACTGGAAAGAAGCG-3′, ParTYO_TPS–TPP-2 reverse: 5′-GATATCTGCAGAATTCCTAGGCGGACACGGCCAG-3′, RvTPS–TPP forward: 5′-TTAAACTTAAGCTTGGTACCATGGTAGTGGAAGACGCG-3′, RvTPS–TPP reverse: 5′-GATATCTGCGGAATTCTTACTGGCTTGAACCAGCC-3′. Amplicons were inserted into pcDNA3.1(+) vector digested with KpnI and EcoRI (Takara, Japan) by using In-Fusion HD Cloning Kit (Clontech, USA). The sequences of insertions were confirmed by Sanger sequencing.

To transfect the expression construct to human cells, we seeded 4 × 10^5^ Hep2 cells in each well of two 6-well cell culture plates (True Line, Nippon Genetics, Japan). One day later, 2.5 µg of plasmid DNA (empty vector, ParTYO_TPS–TPP-2 and RvTPS–TPP; *n* = 4 each) were transfected by using Lipofectamine LTX Reagent with PLUS Reagent (ThermoFisher, USA). After 48 h incubation, transfected cells were washed, trypsinized (TrypLE Express, ThermoFisher, USA) and collected in 1.5 ml tubes. After centrifugation, supernatants were discarded, and cell pellets were immediately frozen by liquid nitrogen and stored at −80°C.

Preparation of cell extracts was performed according to the literature with slight modifications [51]. Briefly, the cell pellet was lysed in 82.5 µl of 0.25 M Na_2_CO_3_ (pH 11) by using BioMasher (Nippi, Japan). After small aliquots (15 µl) were reserved for protein quantification, the remaining was heated at 95°C for 2 h to inactivate endogenous enzymes including trehalase. Then, 35 µl of each sample was mixed with 294 µl of 0.1 M acetate-sodium acetate buffer (pH 5.2) and centrifuged at 12 000 r.p.m. for 5 min. Supernatants were placed into new tubes and used for trehalose quantification.

#### 4.1.5. Whole-genome sequencing and gene prediction

Adult tardigrades were starved for 7 days to allow for full digestion of their gut content. To remove contaminants as completely as possible, the tardigrades were transferred to new clean agar plates at least twice during the starvation and extensively washed with plenty of water prior to DNA extraction. Genomic DNA was extracted from approximately 3000 tardigrades that were extensively cleaned by the procedure described above using Blood & Cell Culture DNA Mini Kit (Qiagen, Germany). Long-read sequencing was performed with Oxford Nanopore MinION system using the Rapid Sequencing Kit (SQK-RAD002) and two R9.4 flow-cells (FLO-MIN106) followed by base calling with Albacore v. 1.0.2 (Oxford Nanopore Technologies, UK). Short reads were produced with Illumina HiSeq 4000 system using TruSeq Nano DNA HT Library Prep Kit (Illumina, USA). Size distribution of the long reads obtained from the Oxford Nanopore platform is shown in electronic supplementary material, figure S4. The sequencing included extremely longer reads (greater than 1 Mbp) than the expected fragment size-range of the DNA extraction kit, which might have been derived from erroneous concatenation during sequencing and were discarded prior to assembly process. As an initial step of assembly, the 7.22 × 10^5^ long reads, ranging from 500 bp to 1 Mbp in length and comprised 7.65 × 10^9^ base pairs in total, were assembled by Canu v. 1.5 with options preserving heterozygous variations (batOptions = -dg 3 -db 3 -dr 1 -ca 500 -cp 50) [52]. The assembled contigs were polished with nanopolish v. 0.6.1 (13 May 2017) [53] using the long reads themselves aligned by minialign v. 0.5.2 [54] followed by a further polish with Pilon v. 1.22 [55] using the Illumina short reads, 7.67 × 10^7^ reads of 1.06 × 10^10^ base pairs, aligned by BWA-MEM v. 0.7.15 [56]. The diploid-aware assembly was processed with HaploMerger2 v. 20161205 [57] to build a haploid assembly, which was again polished with Pilon to generate the final assembly. Quality of the assembly was assessed with BUSCO v. 2.0.1 [58] and CEGMA v. 2.5 [59], both of which were executed on the web tool gVolante [60]. Finally, we obtained the genome assembly of 170 Mb containing 684 contigs (table 1) with the mean average coverages of 45 × and 62 × for the Oxford Nanopore and Illumina sequencing reads, respectively.

Gene model of the *Paramacrobiotus* sp. TYO genome assembly was generated by BRAKER v. 1.11 [61] which includes the gene prediction program AUGUSTUS v. 3.3 [62]. The mapped RNA-seq reads described below for transcriptome analysis were also used as prediction hints (electronic supplementary material, table S7). The BRAKER was executed using the soft-masked *Paramacrobiotus* sp. TYO genome assembly by repeat elements with default parameter settings except for adding the '-filterOutShort' option. The BRAKER run resulted in 41 959 genes and 44 049 transcripts in the genome assembly.

#### 4.1.6. Transcriptome analysis

The *Paramacrobiotus* sp. TYO individuals were starved for 3 days and washed extensively as did for the preparation of the genomic DNA sequencing. We prepared four groups; one hydrated (without exposure to dehydration) and three slowly dehydrated groups for 3 h, 1 day and 2 days. Each group was examined with three replicates except for that of 1-day slow-dehydration treatment that had no replicates and was used for gene prediction only (electronic supplementary material, table S7). The samples, each containing approximately 300 tardigrades, were disrupted in TRIzol reagent (Invitrogen, USA), and total RNA was extracted using Direct-zol RNA MicroPrep Kit (Zymo Research, USA). The libraries were prepared with NEBNext Ultra Directional RNA Library Prep Kit (New England Biolabs, USA). Paired-end sequencing of 150 nt each was performed with Illumina HiSeq 4000, resulting in 14.7 million read pairs on average for the 10 libraries (electronic supplementary material, table S7).

The paired-end reads of the libraries were aligned to the genome assembly by HISAT2 v. 2.0.5 [63], and gene expressions were quantified by StringTie v. 1.3.3b with the options ‘-B -e -G’ [64] employing the gene model for the *Paramacrobiotus* sp. TYO genome described above. Differential expression analysis was performed with edgeR [65] following the format conversion of the read-count data with the python script prepDE.py provided by the StringTie developer. Correction of *p*-values for multiple comparisons was performed with *q*-value [66]. Differential expression analyses for *R*. *varieornatus* and *H. dujardini* were conducted with a similar way. The RNA-seq data of these species were retrieved from NCBI SRA (accession ID, SRP098585) [21].

#### 4.1.7. Retrieval of the metazoan TPS–TPP genes

The searches for TPA–TPP homologues within the amino acid sequence databases were described in detail in electronic supplementary material. We intensively searched for the metazoan TPS–TPP genes within the genome assemblies deposited in the NCBI Whole-Genome Shotgun (WGS) database (electronic supplementary material, table S5), as well as those deposited in the several genome projects. To distinguish the genuine gene in the genome assembly with those from contaminants, this search restricted the TPS–TPP homologues that were split into multiple exons in the genome assemblies and less than 80% nucleotide similarity with any non-metazoan homologues. The aforementioned *B*. *floridae* homologue and the *Botryllus schlosseri* TPS–TPP gene that was annotated as predicted transcripts provided by the *Botryllus schlosseri* genome project (http://botryllus.stanford.edu/botryllusgenome/) met these criteria. Within the *Ophiothrix spiculata* and *Aiptasia* sp. CC7 genome assemblies, we found the putative TPS–TPP loci, which were missing or incompletely predicted in the original gene models. Accordingly, we retrieved the entire sequences of the genes from the genome assemblies employing an on-demand gene prediction as follows. The genome assemblies of *O. spiculata* and *Aiptasia* sp. CC7 were obtained from Echinobase (http://www.echinobase.org/) and the GenBank assembly (accession ID, GCA_001417965.1), respectively. The amino acid sequence of fruit fly *Tps1* (NCBI RefSeq accession ID, NP_608827.1), a TPS–TPP homologue, was queried against these genome assemblies using tblastn. The genomic regions comprising of an array of the tblastn hits, as well as their flanking 10 kb regions at both ends, were retrieved. These genomic regions were subject to gene prediction with Augustus v. 3.3 employing the Protein Profile eXtension (PPX) mode [67], which used a multiple amino acid sequence alignment of the homologs as a hint. For this purpose, we created a multiple alignment of amino acid sequences of the known metazoan TPS–TPP genes.

Additionally, we searched for the metazoan TPS–TPP homologues from the NCBI Transcriptome Shotgun Assembly (TSA) (electronic supplementary material, table S5) as well as the aforementioned de novo transcriptome assembly of the *P. richtersi* ITA population. The homologues were retrieved from these assemblies by querying the fruit fly *Tps1* amino acid sequence with tblastn.

#### 4.1.8. Molecular phylogenetics

We retrieved the homologues of the TPS–TPP, TPS, TPP and trehalose amino acid sequences of all the kingdom as described in the electronic supplementary material. The amino acid sequences of the individual homologue groups were aligned with MAFFT v. 7.305 using the 'L-INS-i' mode [68]. Unambiguously aligned sites were selected by trimAl v. 1.4.1 [69] using the 'gappyout' mode, followed by another trimAl run with the 'automated1' mode. Using the filtered alignment, the phylogenetic tree was inferred by employing the maximum-likelihood (ML) method by RAxML v. 8.2.9 [70] with the options '-f a -m PROTCATAUTO -auto-prot = bic -# 100,' which denote automatic selection of the amino acid substitution model based on the Bayesian information criterion (BIC) and 100 rapid bootstrap replications. We also performed the Bayesian tree inference with ExaBayes [71] by employing two runs in parallel and four chains for individual runs. We used the default parameters of ExaBayes except aforementioned run and chain numbers. The tree inference under the constraints of tree topologies was performed with RAxML with the '-f d -g' option using the same amino acid substitution model as the ML tree inference. Tree topology test between the ML tree and the trees with the topology constraints was performed with consel v. 0.20 [72] following computation of per site log-likelihoods with RAxML.

## Data accessibility

The raw sequencing data have been deposited in the DDBJ Short-Read Archive database under BioProject accession number PRJDB7223. The *Paramacrobiotus* sp. TYO genome assembly was deposited in DDBJ under accession numbers BHEN01000001–BHEN01000684. Other sequence data containing the repeat-masked genome assembly and gene annotations of *Paramacrobiotus* sp. TYO, de novo transcriptome assembly of *Paramacrobiotus richtersi* ITA, 18S rRNA and COI sequences of these *Paramacrobiotus* tardigrades, TPS–TPP sequences of the *Paramacrobiotus* tardigrades, *O. spiculata* and *Aiptasia* sp. are listed in electronic supplementary material, table S8 and deposited in figshare (https://figshare.com/projects/Metazoan_TPS-TPP_gene_identification/36410). Sequences of the three TPS-TPP genes, 18S rRNA and COI of *Paramacrobiotus* sp. TYO were deposited in DDBJ under accession numbers LC637239-LC637243.

The data are provided in electronic supplementary material [73].
